# Host Diversity and Origin of Zoonoses: The Ancient and the New

**DOI:** 10.3390/ani10091672

**Published:** 2020-09-17

**Authors:** Judith Recht, Verena J. Schuenemann, Marcelo R. Sánchez-Villagra

**Affiliations:** 1 Independent Consultant, North Bethesda, MD 20817, USA; 2 Institute of Evolutionary Medicine, University of Zurich, 8006 Zürich, Switzerland; verena.schuenemann@iem.uzh.ch; 3 Paleontological Institute and Museum, University of Zurich, 8006 Zürich, Switzerland; m.sanchez@pim.uzh.ch

**Keywords:** zoonoses, host diversity, mycobacterial diseases, brucellosis, Chagas disease, coronaviruses

## Abstract

**Simple Summary:**

There is a wide variety of diseases caused by bacteria, viruses, and parasites that are transmitted to humans by different routes from other animals. These diseases, known as zoonoses, represent 75% of new or reemerging infectious diseases. There is a considerable impact of these diseases on the economy and health at local and global levels, including zoonotic diseases caused by the ingestion of food and products derived from animals. The wide range of animal species that host these disease-causing organisms include all groups of mammals. Birds are the second significant animal group to act as hosts for zoonoses. Much progress has been made in understanding disease evolution and animal origin, with important contributions from fields such as paleopathology and analysis of DNA, applied to ancient human bone remains. The study of ancient diseases such as brucellosis and tuberculosis benefits from these approaches. More research is needed as new diseases emerge causing pandemics and some previously eradicated reemerge in some regions. Global efforts are focused, based on evidence generated by research, on the prevention of new pandemics.

**Abstract:**

Bacterial, viral, and parasitic zoonotic diseases are transmitted to humans from a wide variety of animal species that act as reservoir hosts for the causative organisms. Zoonoses contribute an estimated 75% of new or reemerging infectious diseases in humans. All groups of mammals have been shown to act as hosts for transmission of different organisms that cause zoonoses, followed in importance by birds; with both wild and domestic species identified as hosts in specific cases. There has been considerable research progress leading to a better understanding of the host range, animal origin, evolution, and transmission of important zoonoses, including those caused by the ingestion of food and products derived from animals. Paleopathology studies of ancient human bone lesions, in combination with ancient DNA analysis of the causative pathogen, have contributed to our understanding of the origin of zoonotic diseases, including brucellosis and mycobacterial zoonoses. However, there are still knowledge gaps and new confirmed and potential hosts are reported locally with some frequency. Both the economic cost and burden of disease of zoonoses are substantial at local and global levels, as reflected by recent coronavirus pandemics that spread rapidly around the world. Evidence-based prevention strategies are currently a global priority increasingly recognized, especially in zoonoses-affected regions.

## 1. Introduction

Zoonoses, or diseases transmitted to humans from vertebrate animals in both rural and urban settings where humans live, are widespread, with an estimated 60% of all human infectious diseases and 75% of new or reemerging infectious diseases considered zoonotic [1]. Zoonoses can be caused by a variety of pathogens, including bacteria, viruses, parasites, or fungi. Of those that infect humans, an estimated 80% of viruses, 50% of bacteria, 40% of fungi, 70% of protozoa, and 95% of helminths are zoonotic [1].

Zoonotic infections result in a wide range of diseases involving diverse hosts and sometimes vectors or parasites with complex life cycles, associated with considerable burden at local as well as global level on both human and animal health, and with great socioeconomic impact on endemic populations. This impact has been recently recognized, leading to global initiatives such as the ‘One Health’ approach, resulting in a holistic consideration of human and animal perspectives, and strong collaboration between environmental health sectors [2]. Early and rapid detection of zoonotic pathogens is crucial to allow a proper response to zoonoses emergence. Unfortunately, tools for this purpose are usually limited in low-resource settings. Disease prioritization may lead to appropriate capacity building and resource allocation in this regard. As a One Health initiative, priority zoonoses workshops were conducted in 2014–2016 in Thailand, Kenya, Ethiopia, Azerbaijan, Cameroon, South Africa, and the Democratic Republic of the Congo, with each country’s list including 37 zoonoses in average and most countries listing the following: (1) bacterial zoonoses: anthrax, brucellosis, leptospirosis, plague, Q fever, salmonellosis, and zoonotic tuberculosis; (2) viral zoonoses: Crimean-Congo hemorrhagic fever, coronaviruses (including MERS and SARS), flaviviruses (including yellow fever and West Nile), hemorrhagic fever viruses (including Ebola and Marburg), rabies, and influenza viruses; (3) parasitic zoonoses: cysticercosis, echinococcosis, and toxoplasmosis [3].

The economic losses and financial costs associated with zoonotic diseases are enormous, for example, avian influenza in Asia in 2004–2009 impacted public and animal health, especially the livestock sector, for an estimated 20 billion US$ total cost; SARS in 2002–2003 disrupted trade and travel in China, with an estimated 41.5 billion US$ cost, and neglected zoonoses affecting poor livestock keepers cause 2.4 billion cases and 2.2 million deaths annually (reviewed in [4]). The most striking and recent example of the worldwide economic impact of a zoonosis is the ongoing COVID-19 pandemic affecting the global economy in “the most serious challenge of the post-war era due to the sudden halt in economic activity in both advanced and developing countries”, which includes global poverty increasing for the first time since 1990 [5].

Despite the facts above, funds allocated for zoonotic diseases have not recently increased. For example, the USA CDC budget in the “Emerging and Zoonotic Infectious Diseases” category has decreased between 2016 and 2018, from 582 to 514 million US$ per year [6]. Every year one in six Americans is estimated to get sick from contaminated foods or beverages and 3000 of them die, with a foodborne illness cost of US$ 15.6 billion annually.

Transmission of zoonotic diseases happens in a variety of ways depending on the human direct or indirect contact with the animal source or related products. A manner of transmission is eating contaminated or undercooked milk, meat, and eggs, as well as raw fruits and vegetables contaminated with livestock excreta. Direct contact with body fluids from infected animals including pets, or getting scratched or bitten, in rabies transmission, for example, also causes zoonotic diseases; indirect contact with animal habitats or contaminated surfaces, or insect vector bites (ticks or mosquitoes) do as well. Some zoonoses need an intermediate vector to be transmitted from vertebrate animals to humans, including the plague (from rats via fleas to humans), Lyme disease (from mice, via ticks), and West Niles virus infection (from birds via mosquitoes). Zoonoses can also occur as a result of leisure activities such as outdoor camping, hiking and caving, visits to zoos and farms, and water activities including jacuzzi, canoeing, and sailing [7].

Diseases that are caused by ingesting contaminated food are of global public health concern, affecting countries worldwide. Especially in low- and middle-income countries, the risk for the zoonotic disease to spread is higher due to farming, slaughtering, processing, and decontamination methods used and weak veterinarian disease control (reviewed in [8]). Some zoonotic pathogens present an additional challenge from a public health perspective: the increasing frequency of antimicrobial-resistant isolates. As the leading cause of zoonotic disease in both animals and humans, *Salmonella* was placed in an antimicrobial resistance (AMR) “serious threats” category of the USA CDC in 2019 [9]. Some strains of *Salmonella* causing infections have developed resistance to antibiotics such as ciprofloxacin, azithromycin, and ceftriaxone, often used to treat patients with severe infections [10]. In the US, *Campylobacter* causes an estimated 1.5 million campylobacteriosis infections annually, of which 29% have decreased susceptibility to antibiotics used to treat these infections, including ciprofloxacin and azithromycin, limiting treatment options especially in low- and middle-income countries [11]. In 2017, a systematic review and meta-analysis of studies on interventions to reduce antibiotic use in food-producing animals compared the presence of antibiotic-resistant bacteria in animals and humans [12]. Based on this review, the World Health Organization (WHO) launched new guidelines on the use of antimicrobials in these animals, recommending to stop using antibiotics routinely to promote growth and prevent disease [13].

Here we present a review on zoonoses host diversity and animal origin of selected zoonoses, with an emphasis on brucellosis and mycobacterial zoonoses. We discuss examples of paleopathology and ancient DNA studies contributing to our knowledge on disease origin and evolution, followed by viral zoonoses including recent pandemics as examples of recent and emerging zoonoses.

## 2. Zoonotic Diseases Host Diversity

The range of animal hosts of pathogens linked to zoonotic diseases is in most cases impressively diverse. Some zoonotic disease outbreak studies have revealed new (sometimes unexpected) animal sources of human infection. Hosts may include both wild and domesticated animals, some leading to foodborne zoonoses. Monitoring animals for the presence of important pathogens is the focus of public health strategies.

Aside from pathogens shared by humans with invertebrates (vectors or intermediate hosts for disease transmission), the majority (about 80%) of the reservoirs known for zoonotic diseases are mammalian, followed by avian hosts [1]. Among other mammalian major taxa (“orders”), humans share the most pathogens with artiodactyls (frequently in proximity), followed by rodents, carnivorans, and primates. Taxonomic identification of source groups for the emergence of zoonotic diseases could help to improve targeting of surveillance and interventions leading to prompt containment and even prevent zoonotic pandemics. However, given the taxonomic breadth reported for the many diseases, pinpointing specific groups and concentrating on them may not be justified. The information on hosts collected in Tables S1–S3 reflects how imprecisely and often very generally this is reported, and how vague our knowledge is in most cases. For example, often reports are of “rodents”—this group alone represents more than one-third of recognized mammalian species, with over 2277 of them [14].

Bats are the natural reservoirs of a significant number of important viral zoonoses, including probably the recent COVID-19 that caused a high impact global pandemic this year (2020), still ongoing and discussed below. It has been hypothesized that bats are unique hosts in this regard, in particular as compared with rodents, the other is a particularly speciose major group (order) of placental mammals besides bats. However, the total number of zoonotic viruses identified in bats (61) was lower than in rodents (68). There is a higher number of rodents than bat species (2-fold) [14,15].

A recent report on the largest dataset of the zoonotic virus—reservoir relationships built with avian and mammalian reservoir hosts of 415 viruses aimed at determining whether some animal species are special reservoirs of zoonotic pathogens [16]. The results did not support this view but a host-neutral variation instead; the analysis showed that the proportion of viruses infecting humans showed minimal variability across reservoir taxonomic orders and were in agreement with the number of animal species within each group (including for bats and rodents), with rare reservoir host effects restricted to one or two viral families.

The camel has been highlighted in a recent review that showed 19 zoonoses reported in this animal in Iran (where raising camels is common with close contact between farmers and camels well as meat and milk consumption) including plague, Q fever, campylobacteriosis, tuberculosis, salmonellosis, rabies, MERS, and toxoplasmosis ([17], Section 4). A plague outbreak in southern Afghanistan in 2007 manifested as 83 cases of acute gastroenteritis with 17 deaths; the outbreak was linked to the consumption or handling of camel meat [18].

Birds are the main reservoir of the influenza A virus (Table S2). Pigs, susceptible to infection with both avian and mammalian influenza viruses, may be acting in interspecies viral transmission as “mixing vessels”, in which avian and mammalian influenza viruses recombine through a process known as “reassortment” to produce novel strains that can then infect humans [19].

A role for wild migratory birds in the transmission of zoonoses has been acknowledged either as a reservoir host or by dispersing infected arthropod vectors. For example, West Nile virus was likely introduced in the USA via infected birds, expanding in 1999–2000 along the Atlantic seaboard, a common bird migration route, to reach southern Florida to establish an enzootic cycle with mosquitoes and then from 2000 to 2002, expanded westward possibly as a result of elliptical avian migration routes (reviewed in [20]). Migrating birds can also carry one or more ectoparasites such as mites, ticks, fleas, and lice, all arthropods that themselves can carry pathogens; a potential role for migrating birds in dispersing ticks and associated pathogens that cause zoonotic disease has been recently highlighted [21].

Gut microbes and obligate ectoparasites such as ticks, fleas, and lice, depending on their animal hosts for transport. In this context, it is remarkable that recent geological events may have had a large impact on the dispersal capacity of both ectoparasites and gut pathogens. The megafauna (animals over 44.5 kg (98 lb) body weight) decline in the Late Pleistocene/early Holocene, which led to a decrease in seeds and nutrients dispersal, may have also caused a reduction in the movement of ectoparasites and generalist fecal microbes, including *Escherichia coli,* to ~15% of pre-extinction levels based on reductions after extinction estimated using the average home range for modern and extinct species [22]. In this study, the distance that gut pathogens can travel between consumption and defecation, which is size-dependent, showed the largest declines in the Americas and Eurasia, however, whether pathogens disappeared with the previous hosts or adapted to new hosts is not known. This subject could be explored by metagenomic analyses of pathogens present in extinct mammal dung [22].

### Chagas Disease

Chagas disease is an example of a successful parasitic zoonosis with a wide range (hundreds) of mammal species hosts in South America, transmitted by dozens of triatomine bug species (Table 1). Known as American trypanosomiasis, it also owes its name to Carlos Chagas, a Brazilian researcher who first described it in Brazil in 1909 as a disease due to the parasite *Trypanosoma cruzi* (named after Oswaldo Cruz, another Brazilian scientist), later shown to parasitize species of mammals from seven “orders” and triatomines from 15 genera [23]. A comprehensive study of reservoirs and wild hosts of *T. cruzi*. in Brazil showed that species of several placental “orders” (Artiodactyla, Chiroptera, Primates, Carnivora, Rodentia, Cingulata, Pilosa) and one marsupial (Didelphimorphia) were involved in transmission, with four of them (Primates, Didelphimorphia, Chiroptera, and the Carnivora species *Nasua nasua*) considered as key reservoir taxa exhibiting higher rates of parasitemia [24]. In Latin America, about 16 million people are estimated to be infected with *T. cruzi* through feces or night bites of the “kissing bug” (triatomines), or through blood transfusion, maternally, or orally through contaminated food that can lead to an acute phase usually without symptoms or very mild [25]. After 10–20 years following the acute phase, however, symptomatic chronic disease can occur, leading to irreversible damage to several organs such as the heart, esophagus, and colon [26].

*Trypanosoma cruzi* has been identified in hosts such as armadillos and monkeys (in 1912 and 1924, respectively, by Chagas in Brazil) [24], as well as cats and dogs. A recent report of a study lasting two decades (1992–2017) of *T. cruzi* infection in wild mammals in Brazil showed that 17% of mammals were seropositive and 8% with high parasitemia, indicating infectivity potential [30]. A recent study reported a potential effect on Chagas disease transmission of oil palm plantations in Colombia, where the main vector in the region has been captured and reported to have *T. cruzi* natural infection, based on vertebrate host analyses of blood meals from nymphs that revealed 18 vertebrate species including pigs, house mouse and opossum [31]. Hunting dogs in indigenous Mayangna and Miskitu populations from Nicaragua’s remote Bosawás Biosphere Reserve were shown to have *T. cruzi* antibodies and therefore previous exposure (7/78 sera screened, or 9%), suggesting hunting dogs as the potential zoonotic risk for Chagas disease in these communities [32].

The origin of Chagas disease is unclear. Bats may have been the original reservoirs hosts of *T. cruzi*, as postulated in the bat-seeding hypothesis, followed by a host switch to a non-volant mammal and then several switches to humans resulting in the diversity of lineages circulating in human populations. Two genetic lineages of trypanosomes associated with bats were recently detected in rural areas of southern Ecuador [33], one of which, named TcBat, is *T. cruzi*-related and was detected in a 5-year-old female in a forest area in northwestern Colombia as a mixed infection of *T. cruzi* I and TcBat genotypes [34].

## 3. Brucellosis and Mycobacterial Diseases: Examples of Ancient Bacterial Zoonoses

A recent systematic review on zoonotic diseases that manifest with human febrile illness reported in 53 malaria-endemic countries showed a wide distribution of these diseases causing febrile illness; half of them were bacterial diseases [35]. For a list of selected bacterial zoonoses reflecting the impressive diversity of this group of diseases, see Table S1. Next, we discuss two well-known bacteria genera—*Brucella* and *Myocabacteria*—as they represent ancient human diseases for which origins are still highly debated.

Two major diseases caused by these bacteria are brucellosis and tuberculosis. They affect the skeleton, causing bone pathologies in about 10–15% of individuals who have the disease [36] and can result in similar lesions in affected individuals despite being caused by very different bacterial species. Paleopathology studies along with ancient DNA analysis of bone lesion remains have been critical in determining the origin for each of these two diseases. The overlapping appearance of affected bones in the spine in both infections have led researchers to initially suspect a tuberculosis lesion, with molecular analyses demonstrating in some cases that the cause was brucellosis. A study that evaluated calcified nodules from a 14th-century skeleton found at an abandoned medieval village in northwest Sardinia, Italy, initially hypothesized the nodules were due to tuberculosis, but shotgun metagenomics revealed medieval *Brucella melitensis* genome sequences instead [37]. Even today, with advanced health technology, there is overlap in the understanding of symptoms and laboratory test results for these two diseases [38], making differential diagnosis difficult in countries such as India, where they are both endemic [39].

### 3.1. Brucellosis

Caused by bacteria of the genus *Brucella* (mostly *Brucella melitensis*), brucellosis is a common zoonotic infection resulting in febrile illness in humans, mainly through the ingestion of unpasteurized dairy products and direct contact with infected animals (Table 1). Travelers to endemic areas may get infected by consumption of unpasteurized milk or other dairy products and may be the source of imported cases into their own countries (mostly from infected cheeses consumed by their families), as is the case for most of the acute brucellosis cases in North America and northern Europe [29].

Re-emergence of zoonotic diseases in countries where they have been previously eradicated has been reported, calling for a need for disease-free countries to remain aware and implement regional monitoring systems. Brucellosis has reemerged in Bulgaria after 50 years, probably due to the illegal import of infected animals from endemic border countries, as has occurred with bovine brucellosis in France, with possible cross-border brucellosis transmission into Europe from middle-eastern countries with the highest incidences of brucellosis worldwide such as Turkey and Syria (reviewed in [40]).

The exact prevalence of both animal and human disease due to brucellosis is not well known. The economic burden of brucellosis is considerably high in low-income countries in tropical Asia and Africa [41]. In 2016, out of 1.27 million estimated cases of brucellosis in Kenya, 12,004 people died, 96% of whom were livestock keepers [42].

Brucellosis can have skeletal manifestations, with bones and joints affected being the most frequent complications occurring in up to 40% of cases [29]. Analysis of vertebral lesions and pathological changes described in a 2.4–2.8 million-year-old male skeleton of *Australopithecus africanus* from South Africa were interpreted as resulting from initial phases of brucellosis [43]. This disease is an example of human and domestic animal paleopathology studies suggesting brucellosis in ancient bone remains, with most cases involving adult male skeletal individuals showing lumbar vertebrae and sacroiliac joints involved [44], evidence which combined with ancient DNA analysis by PCR have confirmed the presence of *Brucella* DNA (reviewed in [45]). As discussed above, DNA detection of *Brucella* DNA has been critical in confirming brucellosis in ancient human remains when paleopathology initially suggested tuberculosis.

However, *Brucella* bacteria do not preserve as well as mycobacteria, which have a thicker, hydrophobic and more resistant cell wall that has allowed more data to be collected for ancient infection for the latter type of disease-causing bacteria (discussed next).

### 3.2. Mycobacterial Zoonoses

Mycobacteria vary in epidemiology, reservoirs, and their ability to cause disease, with groups such as the *Mycobacterium tuberculosis* complex including *M. tuberculosis* and *M. bovis* (see bovine tuberculosis below) that infect a large range of mammals including humans via inhalation of droplets containing bacteria that reach the lungs; *M. leprae* (see leprosy section below); “nontuberculous mycobacteria” [46].

Human tuberculosis caused by *M. tuberculosis* is a well-known disease of unclear origin, with a considerable impact on global health. It affects an estimated 10 million people annually. Those living with HIV are more likely to be infected with and die of tuberculosis [47]. The origin of tuberculosis is under debate regarding whether it started as a zoonosis from cattle or not. One hypothesis states that it originated with bovid milk consumption known to occur in the early Neolithic in Europe [48]. Another hypothesis based on biomolecular studies proposes a new evolutionary scenario with human tuberculosis having a human origin, present in early African human populations at least 70,000 years ago and expanding with human migration, especially in the Neolithic [49]. In both scenarios, human-animal proximity starting in the Neolithic was a critical factor for emergence/expansion, due to close interaction between early domesticates and humans and increased human density. Human density in some areas peaked during the industrial revolution when crowded dairy cow populations in densely populated urban settings were the source of milk [48].

Although rare, tuberculosis can result in osteological lesions which can help identify, along with molecular analysis, tuberculosis infection in ancient bone remains. These approaches have been applied to materials from both humans and animals. However, molecular genetics applications in these cases have limitations, including cost. Human remains from a medieval churchyard in England in which both *M. tuberculosis* and *M. bovis* were predicted due to milk and beef consumption at the time, showed only *M. tuberculosis* DNA in human skeletons that displayed morphological symptoms of tuberculosis (reviewed in [48]).

A particular question on ancient DNA in this context relates to the origins of tuberculosis in the Americas. While today European lineages of *M. tuberculosis* are found, morphological evidence exists that may support a pre-Columbian prevalence of the disease. A recent report on sequencing and analysis of three mycobacterial genomes from Peruvian human skeletons dated to 1028–1280 revealed the presence of tuberculosis before European contact in pre-Columbian South America [50], a region with considerable morphological evidence of pre-Columbian tuberculosis previously reported. In this study, the three sequenced mycobacterial genomes were most closely related to bacteria belonging to the *M. tuberculosis* complex that have adapted to seals and sea lions (*M. pinnipedii*) than those adapted to humans today in Europe, Asia, or Africa. These data suggest that sea mammals may have played a role in tuberculosis transmission to humans across the ocean, an intriguing possibility for a zoonotic origin of New World tuberculosis to be further investigated.

#### 3.2.1. Bovine Tuberculosis

Bovine tuberculosis resulting from *M. bovis* infection affects cattle and other mammals and, when transmitted to humans, becomes zoonotic tuberculosis (Table 1). Its overall incidence has decreased due to cattle control and routine pasteurization of milk from cows, as infection happens mostly by consuming unpasteurized milk and dairy products or by wound contact occurring during slaughter or hunting [51]; occupational exposure of livestock workers may occur via inhalation of aerosol or cough from infected cattle.

This form of widespread zoonotic tuberculosis can be fatal, present mainly in Africa (causing approximately 3% of all pulmonary tuberculosis cases) and southeast Asia. In 2016, out of 9689 cases of bovine tuberculosis in Kenya, 1168 people died (about 12%); 70% of the fatalities were livestock keepers [42]. Often bovine tuberculosis caused by *M. bovis* in humans is indistinguishable clinically from human tuberculosis resulting from *M. tuberculosis* infection; bovine tuberculosis is estimated to account for up to 10% of human tuberculosis cases in some countries [52]. However, accurate information is lacking on the incidence of tuberculosis due to human *M. bovis* infection from countries with high tuberculosis and HIV prevalence and where populations have direct contact with cattle such as sub-Saharan Africa [53,54]. In these low resource settings, it is often assumed (and not tested) that tuberculosis is caused by *M. tuberculosis*, leading to underestimating the real incidence of zoonotic tuberculosis and underscoring a need to accurately diagnose and treat human tuberculosis caused by *M. bovis* [55].

*M. bovis* has a broad host range including domestic and wild animals, with feral maintenance hosts such as badgers (*Meles meles*) in the UK and Ireland, the brushtail possum (*Trichosurus vulpecula*) in New Zealand, and the white-tailed deer (*Odocoileus virginianus*) in Michigan, USA posing a threat for livestock infection as well as disease eradication from cattle (reviewed in [30]). Cases of bovine tuberculosis in humans linked to deer hunting and handling in an endemic white-tailed deer area have been reported in Michigan [56]. There is also epidemiological evidence in New Zealand that feral ferrets (*Mustela furo*) may be tuberculosis vectors for cattle, and can be used as sentinels for this disease as an alternative to possums (reviewed in [57]).

#### 3.2.2. Avian Tuberculosis

*Mycobacterium avium*, a causative bacterium of tuberculosis in birds, can also be transmitted to mammals, including livestock. The livestock industry suffers economic losses and trade restrictions due to its incidence. Widespread paratuberculosis in animals (also known as Jones disease) including bovine paratuberculosis, is an endemic disease especially in developing countries affecting livestock production, zoo, and wildlife animals (reviewed in [58]). *M. avium* subspecies can cause paratuberculosis in humans, manifested as inflammatory bowel disease and autoimmune diseases including asthma, insulin-dependent diabetes mellitus, sarcoidosis, rheumatoid arthritis, multiple sclerosis, celiac disease and may also be a contributing factor to Crohn’s disease [58].

#### 3.2.3. Leprosy

*Mycobacterium leprae*, the main causative agent of leprosy in humans, a chronic disease with skin lesions and peripheral nerve damage mostly spread via a human-to-human transmission with zoonotic transmission from natural reservoirs, which are not clearly understood yet. The first animal reservoir to be discovered was the nine-banded armadillo (*Dasypus novemcinctus*) in the southern United States [59,60,61]. This armadillo, which can be naturally infected with *M. leprae* and shows similar disease presentation as humans systemically, have been used as animal models for leprosy studies by laboratory infection; they present typical plantar ulceration with foot ulcers increasing as the infection progresses (reviewed in [62]). This species has been recently shown to be a potential reservoir in Brazil, where in some areas people hunt and eat them as a dietary source of protein [63].

Recently, *M. leprae* was also isolated from red squirrels on Brownsea Island in the southern UK [64]. For both nine-banded armadillos and red squirrels reservoirs, related *M. leprae* strains were found via ancient DNA studies in human skeletons from England and Denmark dating to medieval times, suggesting a potential European origin of the strains present today in the reservoirs [65,66].

Non-human primates in several regions have been identified as additional *M. leprae* reservoirs [67]. *M. leprae* genomes were obtained from a chimpanzee (*Pan troglodytes*) from Sierra Leone, a cynomolgus macaque (*Macaca fascicularis*) from the Philippines, and a sooty mangabey (*Cercocebus atys*) from West Africa [67]. Marmosets (*Callithrix jacchus*) in Brazil, also examined as possible hosts for *M. leprae,* were not positive by DNA analysis although mycobacterial DNA (rpoB1 locus) was detected [68].

## 4. Wild Animals and Recent Viral Zoonoses

In contrast to the previously discussed zoonoses, which are linked to well-known diseases, viral zoonoses are the cause of the majority of recent human pandemics, suggesting that viruses may evolve more rapidly than other pathogens to adapt to the human host, sometimes leading to the person-to-person transmission without the need for another reservoir, and with transmission enhanced in dense populations and by human travel [1]. Many of them are distributed worldwide (Table S2). Here we present examples of recent viral zoonoses, some resulting in widespread pandemics, to showcase the clear involvement of wild animals, which are often debated in the previously introduced cases likely due to the old nature of those diseases.

In many areas of central Africa in particular, wild meat (known as bush meat) is in high demand. Human contact with wild animals during hunting and preparation or consumption of undercooked meat has led to important zoonoses developing. An example may be HIV/AIDS, which was linked to the butchering of hunted chimpanzees in Africa, afterward adapting to human-to-human transmission [69]. The human Ebola epidemic in West Africa in 2014–2016 caused by the Ebola virus was linked to the hunting or handling of infected gorillas and other wild animal carcasses ([70], Table S2). The Congo’s Ebola hemorrhagic fever affects gorillas and chimpanzees; about 5000 gorillas were killed in Gabon and the Republic of the Congo in 2002–2003 by the Ebola virus [71].

Asia is another example of wild animals trafficking for consumption, especially in China, Myanmar, Vietnam, and Thailand, where “ye wei” (“wild taste” for wild and exotic animals) was associated with social status including in the dynastic eras as well as currently being widely sold in wet markets and restaurants, sourced legally or illegally from the wild or wildlife farms (reviewed in [72]). Important zoonoses such as some caused by coronaviruses (Table 2) have also been associated with the consumption of wild meat in markets, two of them in China. The severe acute respiratory syndrome coronavirus (SARS-CoV) in 2003 and the Middle East respiratory syndrome coronavirus (MERS-CoV) in 2012 each caused a large pandemic, but these were small compared to the one caused by SARS-CoV-2, a second SARS virus recently identified as the cause of COVID-19. The SARS pandemic caused 8098 cases and 774 deaths and MERS resulted in 2494 cases and 858 deaths [73], while COVID-19 has caused, as of 4 July 2020 and only six months after WHO declared it a pandemic on 11 January, over 11 million cases and slightly over half a million deaths [74].

The animal sources for SARS and MERS were identified as the civet and dromedary camel, respectively (Table 2). However, these are considered intermediate hosts, as these zoonotic diseases are thought to have originated in bats [75,76], subsequently spilling over to intermediate hosts, and eventually jumping to humans [75]. A very recent phylogenetic dating study based on bioinformatic approaches strongly suggests that SARS-CoV-2 emerged directly, without an intermediate host, from the same horseshoe bat subgenus of SARS-like coronaviruses, with both SARS-CoV and SARS-CoV-2 diverging at the same time (40–70 years ago) from currently known extant bat virus [77].

Nonhuman primates were shown to be an effective yellow fever sentinel system in Brazil. Yellow fever (Table S2), a reemerging viral zoonotic disease endemic in Africa and South America transmitted from vector mosquitoes, often causes outbreaks in both humans and nonhuman primates in Brazil. This country has an established and successful yellow fever national surveillance program that includes postmortem nonhuman primate studies for early circulating virus detection and prompt implementation of vaccination and vector control [78]. After a 2017 epizootic occurred in Brazil’s Espirito Santo state, 22 deceased nonhuman primates (two howler monkeys (*Alouatta* spp.) and 20 not further identified) were examined with 21 of them showing typical yellow fever features; yellow fever was diagnosed the same year for 150 of 1000 animals tested from southern states of Brazil (15% occurrence) [79].

Emerging zoonotic diseases causing epidemics have prompted scientists to focus on efforts towards identifying factors involved in disease emergence as well as possible prediction tools, including modeling approaches. Recent such modeling used for spatial mapping of hotspots showed that the global distribution of the risk of emerging zoonotic diseases is higher in tropical regions with wildlife biodiversity (especially mammals) experiencing land-use changes [80]. This study, along with previous ones [81], predicted a higher risk in tropical, developing countries. The authors stated that even though emerging infectious disease events have been predominantly reported in developed countries, this is probably an artifact due to stronger surveillance and reporting systems in these areas. It would be hard to test this idea.

The One Health multisector approach response to zoonoses threat is an appropriate one, which may need input from additional sectors, such as travel and tourism, social and political scientists, anthropologists, and economists to better plan and implement strategies related to surveillance, capacity building, and risk reduction when addressing, for example, the possible closure of wet markets in communities that rely heavily on them for income and food [82].

## 5. Conclusions

There is a wide range of species reported as hosts of zoonoses, making predictions on new and potential reservoirs based on phylogenetic considerations challenging. Basic information on which species serve as hosts is lacking for many regions and diseases. Although considerable progress has been made in our understanding of these diseases’ reservoirs, animal origin, and evolution, with contributions made by paleopathology and ancient DNA detection for ancient diseases such as brucellosis and zoonotic tuberculosis. Further research is needed to gain insights into mechanisms of disease emergence and transmission. In view of the recent pandemics, research on zoonoses should be prioritized towards developing evidence-based prevention strategies. These should include approaches based on sustainable living and food consumption that address inequality, as commentators across the globe currently discuss prompted by the COVID-19 pandemic.

## Figures and Tables

**Table 1:** Selected zoonoses reviewed *.

Disease	Causative Pathogen	Region	Main Reservoirs	Mode of Transmission to Humans
Chagas disease	*Trypanosoma cruzi*	Southern USA, Central and South America	Opossums, rodents, armadillos, dogs, cats, and other mammals including monkeys (7 orders of mammals)	Contact with the fecal material of Triatominae bug, ingestion of contaminated food; blood transfusion
Brucellosis	*Brucella* spp. (*B. abortus, B. melitensis, B. suis, B. canis, B. pinnipedialis*, and *B. ceti*	Worldwide	Cattle, bison, water buffalo, African buffalo, elk, deer, sheep, goats, camels, swine, and wild pigs (*B. suis*); dogs and wild canids (*B. canis*); marine mammals (*B. pinnipedialis* and *B. ceti*)	Ingestion of unpasteurized dairy products or undercooked meat, contact with mucous membranes, and broken skin
Tuberculosis (bovine)	*Mycobacterium bovis*	Previously worldwide, now mostly eradicated or rare (Africa and Southeast Asia)	Cattle, bison, African buffalo, cervids, brushtail possums, badgers, kudu can be reservoirs	Ingestion (unpasteurized dairy products, undercooked meat including bushmeat), inhalation, contamination of breaks in the skin

* Information sources: [23,25,27,28,29,30]; additional tables with information on many bacterial, viral, and parasitic zoonoses are in the supplementary material (Tables S1–S3, respectively).

**Table 2:** Coronavirus zoonoses *.

Disease	Causative Pathogen	Region	Main Reservoirs **	Mode of Transmission to Humans
Severe acute respiratory syndrome (SARS)	SARS-CoV coronavirus	China origin in 2002, with spread to southeast Asia	Civet	Direct and indirect contact, respiratory droplets
Middle East Respiratory Syndrome (MERS)	MERS-CoV coronavirus	Middle East (Arabian Peninsula, origin in Saudi Arabia in 2012) with some travel spreading to other countries	Dromedary camels	Probably direct and indirect contact
COVID-19	SARS-CoV-2 coronavirus	China origin in 2019, gradually spreading globally in early 2020	Undetermined, probably wild animals sold at the market	Probably direct and indirect contact

* Diseases information sources: [27,28,73,74,75] ** For all these zoonoses, a bat is thought to have been the original host, a hypothesis that requires further testing.

## Supplementary Materials

The following are available online at https://www.mdpi.com/2076-2615/10/9/1672/s1. Table S1: Selected bacterial zoonoses, Table S2: Selected viral zoonoses, Table S3: Selected parasitic and other non-bacterial non-viral zoonoses.
