# Supplementary material for: Host Diversity and Origin of Zoonoses: The Ancient and the New
Source: Animals (Basel). 2020 Sep 17;10(9):1672. doi: 10.3390/ani10091672 (PMC7552289; doi:10.3390/ani10091672)
Supplement: Supplementary file 1 [file animals-10-01672-s001.pdf]

Table S1. Selected bacterial zoonoses *.

Disease	Causative Pathogen	Region	Main Reservoirs	Mode of Transmission to Humans
Anthrax	*Bacillus anthracis*	Africa, Asia, South America, Middle East, parts of Europe	Cattle, sheep, goats, horses (most mammals), some birds	Occupational contact exposure, ingestion/foodborne, rarely airborne
Campylobacteriosis	*Campylobacter* spp	Worldwide	Poultry, farm animals, wild birds	Foodborne (raw meat, milk) and direct contact with infected animals (fecal/oral)
Chlamydiosis	*Chlamydia abortus*, *C. felis*	*C. felis* worldwide; *C abortus* in most sheep-raising areas (not Australia or New Zealand)	*C. abortus* in sheep, goats, cattle, other mammals; *C. felis* in cats	Contact with animals
Enterohemorrhagic *Escherichia coli* infections	*E. coli* O157:H7 and other types/serogroups	Worldwide	Cattle, sheep, goats, bison, deer, pigs, other mammals, birds	Ingestion of undercooked meat (ground beef), vegetables, or contaminated water; direct contact with feces or contaminated soil
Leptospirosis	*Leptospira* spp.	Worldwide	Reservoir hosts include rodents, dogs, cattle, pigs, farmed red deer, others	Occupational and recreational exposure, or exposure to urban rodent-contaminated material, especially skin, mucous membrane contact; water- and foodborne
Listeriosis	*Listeria monocytogenes*	Worldwide	Cattle, sheep, soil	Foodborne via unpasteurized dairy, raw meat, fish, vegetables, contaminated water or soil, direct contact with animals and nosocomial
Lyme disease	*Borrelia burgdorferi*	North America, cases reported in Europe, Australia, parts	Ticks, rodents, sheep, small mammals	*Ixodes* spp. bite

		of Asia, and the Amazon		
Methicillin-resistant *Staphylococcus aureus* (MRSA) infections	*S. aureus* with *mecA* gene; some strains maintained in animals or people but animals can become carriers	Worldwide	Pigs; cats and dogs mainly acquire strains from people; other mammals (horses, cattle); birds and poultry	Usually by direct contact with asymptomatic carrier animals; other routes also described; can be nosocomial in hospitals
Plague	*Yersinia pestis*	Foci in North and South America, Asia, Middle East, and Africa	Rodents and their fleas; some mammals including cats	Flea bite, scratches or bites from infected cats, handling infected animals or tissues
Q (query) fever	*Coxiella burnetti*	Worldwide	Cattle, sheep, goats, cats, dogs, birds, and possibly capybara and kangaroos	Mainly aerosol, direct contact with and exposure to the animal placenta and birth tissues and excreta
Salmonellosis	*Salmonella* spp.	Worldwide	Poultry, cattle, pigs, widespread in mammals, birds, reptiles, amphibians	Direct animal contact, raw meat, food, and fecal-oral infection
Streptococcal infections	*Streptococcus* spp.	Worldwide	Swine, horses, dogs, cats, fish	Ingestion of unpasteurized dairy products, pork; direct contact often through broken skin; *S pyogenes* can colonize bovine udder and be transmitted in milk
Yersiniosis	*Yersinia pseudotuberculosis, Y. enterolitica*	Worldwide	Many domestic and wild mammals, pigs are the main reservoir	Ingestion of raw or undercooked pork, fresh milk, and dairy
Zoonotic diphtheria	*Corynebacterium ulcerans*	Probably worldwide, human incidence may be increasing	Cattle, farm animals, dogs, cats, ferrets	Direct contact, unpasteurized milk consumption

* Diseases information sources: Merck Veterinary Manual [28]; GOV.UK [29]

Table S2. Selected viral zoonoses *.

Disease	Causative Pathogen	Region	Main Reservoirs	Mode of Transmission to Humans
Avian and Swine influenza A	Genus *Influenzavirus A*; swine H3N2, H1N1, avian H5N1, H7N9	Worldwide	Wild and domestic birds, especially poultry; pigs and turkeys	Usually, contact with infected animals shed in respiratory secretions
Buffalopox virus infection	Vaccinia virus, Buffalopox strain (genus *Orthopoxvirus*)	Indian subcontinent (South Asia), Egypt, Indonesia	Water buffalo, cattle	Skin contact with infected animals, often when milking
Cowpox	Cowpox virus genus *Orthopoxvirus*	Parts of Europe and Asia	Rodents, wild and domestic cats	Direct contact (broken skin, bites, scratches)
Crimean-Congo hemorrhagic fever	Crimean-Congo hemorrhagic fever virus genus *Nairovirus*	Africa, Middle East, Central Asia, Southeastern Europe, it may be spreading	Livestock (cattle, sheep, goats), some birds, ticks	Tick bites, direct contact with animal blood, unpasteurized milk ingestion
Ebola hemorrhagic fever	Ebola virus	Africa	Bats are suspected reservoir, primates	Contact with infected primate tissues, possible transmission from cave bats
Foot-and-mouth disease	Foot-and-mouth disease virus (genus *Aphthovirus*)	Asia, Africa, Middle East, South America	Cattle, swine, sheep, goats, others	Contact exposure
Hantavirus syndromes	Hantaviruses	The Americas, Europe, Asia	Rodents, maybe insectivorous species (shrews and moles), bats	Aerosolized excreta, bites, and direct contact of human broken skin with rodents
Hepatitis E	Hepatitis E virus	Worldwide	Pigs, wild boar, deer, maybe camels	Undercooked animal meat consumption, fecal-oral spread
Lassa fever	Lassa virus (genus *Arenavirus*)	West Africa	Wild rodents (multimammate rat)	Contact with rodent excretions, secretions, or tissues; aerosol
Nipah virus infection	Nipah virus (genus *Henipavirus*)	Malaysia, Bangladesh, and northern India	Fruit bats, sometimes swine	Direct contact with contaminated pigs or infected tissue, bat-to-human transmission (contaminated juice, date palm sap)
Rabies	Genus *Lyssavirus* and related lyssaviruses	Worldwide with some exceptions	Dogs, foxes, bats, cats	Bites of diseased animals; aerosols in closed environments
Rift Valley fever	Rift Valley fever virus genus *Flavivirus*)	Africa, foci in Arabian peninsula, Indian subcontinent	Cattle, goats, sheep, buffalo, camels, nonhuman primates, squirrels, puppies, and kittens	Mosquito bites, contact with tissues

West Nile fever and virus infection	West Niles virus (genus *Flavivirus*)	East and West hemispheres	Mostly birds	Mosquito bite (*Culex* spp.)
Yellow fever	Yellow fever virus (genus *Flavivirus*)	South America and Africa	Non-human primates	Mosquito bite

*Diseases information sources: Merck Veterinary Manual [28]; GOV.UK [29] For coronavirus zoonoses see Table 2.

Table S3. Selected parasitic and other non-bacterial non-viral zoonoses *.

Disease	Causative Pathogen	Region	Main Reservoirs	Mode of Transmission to Humans
Onchocerciasis	*Onchocerca* Spp.	Species-specific; mostly Europe, the USA, and Japan	Cattle, horses, cervids, wild boars, dogs and other canids, camels	Probably transmitted by black flies
Pork tapeworm disease; cysticercosis and neurocysticercosis	*Taenia solium, Taenia* spp.	Worldwide where swine are reared; mostly Africa, Asia, Central and South America	People, pigs, cattle, and other mammals as intermediate hosts	Ingestion of undercooked pork with larvae causes taeniasis; ingestion of eggs (including autoinfection from an adult worm in the intestine) causes cysticercosis
Toxoplasmosis	*Toxoplasma gondii*	Worldwide	Domestic cats; other mammals including livestock, deer, and birds considered intermediate hosts	Ingestion of oocysts in feces of infected cats (including contaminated soil, food, water) or tissue cysts in undercooked meat or unpasteurized milk
Variant Creutzfeldt-Jakob (mad cow) disease	Bovine spongiform encephalopath y prion	Most cases in the UK	Cattle and other ruminants, cats and other felids, lemurs	Ingestion of bovine products contaminated with CNS tissues

* Diseases information sources: Merck Veterinary Manual [28]; GOV.UK [29]
